# Protein Interaction Network Analysis to Investigate Stress Response, Virulence, and Antibiotic Resistance Mechanisms in *Listeria monocytogenes*

**DOI:** 10.3390/microorganisms11040930

**Published:** 2023-04-03

**Authors:** Robert Hanes, Fangyuan Zhang, Zuyi Huang

**Affiliations:** Department of Chemical Engineering, Villanova University, Villanova, PA 19085, USA

**Keywords:** antibiotic resistance, bacterial stress response, protein interaction network, *Listeria monocytogenes*, virulence

## Abstract

*Listeria monocytogenes* is a deadly and costly foodborne pathogen that has a high fatality rate in the elderly, pregnant women, and people with weakened immunity. It can survive under various stress conditions and is a significant concern for the food industry. In this work, a data analysis approach was developed with existing tools and databases and used to create individual and combined protein interaction networks to study stress response, virulence, and antimicrobial resistance and their interaction with *L. monocytogenes*. The networks were analyzed, and 28 key proteins were identified that may serve as potential targets for new strategies to combat *L. monocytogenes*. Five of the twenty-eight proteins (i.e., sigB, flaA, cheA, cheY, and lmo0693) represent the most promising targets because they are highly interconnected within the combined network. The results of this study provide a new set of targets for future work to identify new strategies to improve food preservation methods and treatments for *L. monocytogenes*.

## 1. Introduction

The Centers for Disease Control and Prevention (CDC) estimate that there are approximately 48 million cases of foodborne illnesses per year in the United States. Listeriosis, while not common, is one of the leading causes of death from foodborne illnesses [1]. In the U.S., there are approximately 1600 infections per year that result in about 260 deaths, corresponding to a hospitalization rate of 94% and a mortality rate of 16% [2]. The fatality rate can be as high as 30% in the elderly, pregnant women, and people with weakened immunity [3]. *Listeria monocytogenes*, the pathogen that causes listeriosis, has the third highest mortality rate for foodborne pathogens in the U.S.

*Listeria monocytogenes* is a facultative intracellular pathogen that can survive a wide range of stress conditions [4]. It has been found to be a highly occurring pathogen in several countries, including the United States, United Kingdom, Australia, Canada, and Mexico [5]. It is found in the environment and is carried by animals [6]; humans are primarily infected with the bacteria from contaminated foods and surfaces [6]. For these reasons, *L. monocytogenes* is one of the most concerning pathogens for the food industry [7].

Bacterial stress response is a microorganism’s ability to respond to external stresses by expressing proteins that aid in survival. Food preservation methods control the presence and growth of bacteria in the food chain by employing different types of stresses, e.g., thermal stress, acidic stress, osmotic stress, and oxidative stress [4]. *Listeria monocytogenes* is difficult to control in foods because it can survive in low moisture, high salt concentrations, and refrigerated conditions [8]. Understanding the key stress response proteins can lead to the development of more effective food preservation methods and thereby reduce the risk of exposure to *L. monocytogenes*.

Virulence is a microorganism’s ability to cause disease through the expression of virulence factors, i.e., proteins, that help bacteria to invade host cells, evade host defenses, and cause diseases [9]. These include polysaccharide capsules that surround the outside of the pathogen to protect it; surface components such as flagella (protein appendages) that propel the pathogen to move within a host cell; adhesions (extracellular-bound proteins) that enable the pathogen to interact with a host cell; exotoxins and enterotoxins secreted by the pathogen; and Type III secretion systems (an assemblage of proteins) that help the pathogen to secrete proteins into the host cell [10]. For example, in *Listeria monocytogenes*, the proteins plcA and hly are known virulence factors that aid in the escape of the microorganism from the host cell vacuole [11]. Disrupting the expression of virulence factors could lead to fewer infections and better outcomes for patients who are exposed to *L. monocytogenes*.

Antibiotic resistance is a microorganism’s ability to defeat the drugs designed to kill it [12]. Antibiotic resistance is a serious public health issue that is estimated to be a leading cause of death worldwide after stroke and heart disease [13]. In 2019, the CDC reported nearly three million infections and more than 35,000 deaths due to resistant microorganisms [12]. In Europe, such infections were responsible for more than 426,000 illnesses and 33,000 deaths in 2019 [14]. *Listeria monocytogenes* is susceptible to a wide range of antibiotics active against Gram-positive bacteria, except cephalosporins and fosfomycin, for which it has inherent resistance [15]. The most common treatment for listeriosis is ampicillin, used alone or in conjunction with gentamicin [15]. Although the presence of ampicillin-resistant genes is not yet observed to be increasing in *L. monocytogenes* [16], this is an ongoing risk due to lateral gene transfer in bacteria [17]. The identification of new targets to combat antibiotic resistance will ensure that effective treatments continue to be available for infected patients.

Previous studies have looked at the relationships between stress response and virulence. For example, sigB is known to play a role in both stress response and virulence [18]. It has also been shown that there can be an interaction between virulence and antibiotic resistance. For example, *Listeria monocytogenes* can be susceptible to fosfomycin, despite having intrinsic resistance, due to the expression of the virulence genes *prfA* and *hly* [19]. In this work, stress response, virulence, and antibiotic resistance are studied together. First, a method is described to generate protein interaction networks using readily available tools and resources in systems biology. The method is used to create individual and combined protein interaction networks for stress response, virulence, and antimicrobial resistance for *L. monocytogenes*. Lastly, the networks are analyzed to identify key proteins.

## 2. Materials and Methods

A data analysis approach was developed with existing tools and databases to create protein interaction networks. The first step is to generate a list of proteins related to the biological process of interest, e.g., stress response, virulence, and antibiotic resistance. There are several databases available that can be used to generate protein lists, including but not limited to Genemania [20], DisGeNet [21], UniProt [22], and Gene Expression Omnibus (NCBI-GEO) [23]. From the protein list, a network is created and visualized using the tools STRING and Cytoscape. STRING is a database of protein–protein interactions [24] and Cytoscape is a multi-platform network visualization and analysis tool [25]. Both Cytoscape and STRING have been previously used successfully for network development and analysis, for example, to create a gene interaction network to study antibiotic resistance mechanisms in *Proteus mirabilis* [26].

In a protein interaction network the nodes correspond to proteins and the edges correspond to known or predicted protein interactions. The network can be manually curated to add or remove nodes and edges based on published results or other criteria, such as clustering analysis. Generally, nodes that are not highly interconnected may be removed. The network is analyzed based on the topological features of the network to identify key nodes. The functions of the proteins that correspond to the key nodes can be further studied using tools such as the functional enrichment analysis in STRING and the online resource DAVID (Database for Annotation, Visualization, and Integrated Discovery). 

DAVID provides a comprehensive set of functional annotation tools for investigators to understand the biological meaning behind large lists of genes [27].

**Figure 1:**
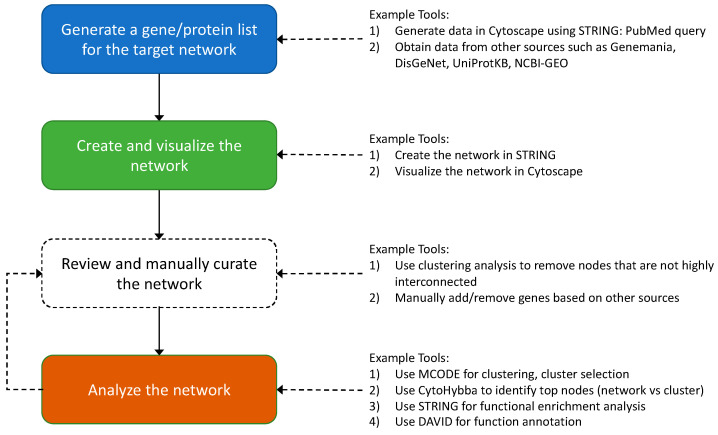
Overview of protein network development and analysis.

The general workflow to create a protein interaction network is summarized in Figure 1. The specific workflow used for this work can be provided upon request.

STRING is a database of known and predicted protein–protein interactions that includes both physical and functional protein associations. The STRING database currently covers 24,584,628 proteins from 5090 organisms [24]. STRING generates a network from an input list of proteins based on associations from a variety of data sources including genomic context predictions, high-throughput lab experiments, automated text mining, and previous knowledge in databases [24]. The network can be viewed within STRING or exported for visualization and analysis outside of STRING; for example, the network can be exported directly to Cytoscape.

Cytoscape is a software platform for visualizing complex networks and integrating attribute data [28]. A network can be imported into Cytoscape from a variety of sources. In addition, a network can be generated within Cytoscape. For example, various types of queries can be performed using the STRING application in Cytoscape to generate a protein list and protein network. The functionality of Cytoscape can be extended through a wide range of applications supporting a variety of problem domains that can be downloaded and managed directly in the software. 

For this analysis, STRING and Cytoscape were used to generate and visualize protein networks for stress response, virulence, and antibiotic resistance. Three individual protein–protein interaction networks were generated in Cytoscape. The STRING: PubMed query function was used to generate the initial protein lists for each network. The settings used to generate the protein networks are listed in Table 1.

There are several options for the data source in Cytoscape. “STRING: PubMed query” was selected to return a STRING network based on a protein list generated from a PubMed query with the specified search term for each network. This resulted in a list of proteins from the PubMed database by using the specified search term, creating the protein interaction network using the STRING database, and displaying the network in Cytoscape. STRING has two species options for *Listeria monocytogenes*. *Listeria monocytogenes* EGDe was selected because it is a commonly used laboratory reference strain [29].

There were no changes made to the default settings for the STRING parameters. “Full STRING network” was selected because it returns both functional and physical protein associations. STRING ranks associations from lowest to highest based on the strength of the supporting data. For this analysis, the confidence score cutoff was set to 0.4, which returns associations that are of a medium-to-highest confidence score. The maximum number of proteins was set to 300. These two settings were selected to ensure that the initial networks included a large number of proteins for the subsequent analysis. In all three cases, the maximum number of proteins, i.e., 300 proteins, was identified and used to create the initial network.

The application Molecular Complex Detection (MCODE) is a clustering algorithm that identifies densely connected regions in a protein interaction network that may represent molecular complexes [30]. The MCODE application was used within Cytoscape to manually curate the networks by removing nodes that were not part of a cluster. The settings used for MCODE are listed in Table 2.

There were no changes made to the default settings in MCODE. Loops were not included in the neighborhood density calculation. The degree cutoff was set to 2, meaning only nodes with two or more connections would be scored. The haircut option was selected so that nodes connected to a cluster by only one edge were removed. Fluff was set to No, ensuring that nodes would only belong to one cluster. The node score cutoff, which determines which nodes to include in a cluster, was set to 0.2. This setting can also be adjusted after the results are generated to change the cluster size. The K-score which determines the minimum number of connections within a cluster was set to 2, resulting in clusters with two or more connections. The maximum depth was set to 100 to avoid arbitrarily limiting the cluster size.

The application CytoHubba scores the nodes within a network based on its topological characteristics [31]. There are two settings in CytoHubba: one to specify the number of nodes to be ranked and one to identify the ranking method. There are eight algorithms available in CytoHubba that can be used to rank the nodes based on various features of the network: MCC (Maximal Clique Centrality), DMNC (Density of Maximum Neighborhood Component), MNC (Maximum Neighborhood Component), Degree, EPC (Edge Percolated Component), Bottleneck, EcCentricity, and Closeness. CytoHubba was used to identify the most highly connected nodes for each network. For this analysis, the top 25 nodes were ranked to avoid arbitrarily limiting the number of nodes returned. The MCC algorithm was used as the ranking method based on prior work that determined that MCC identified more essential proteins compared to the other methods [31].

## 3. Results

### 3.1. Stress Response Network

**Figure 2:**
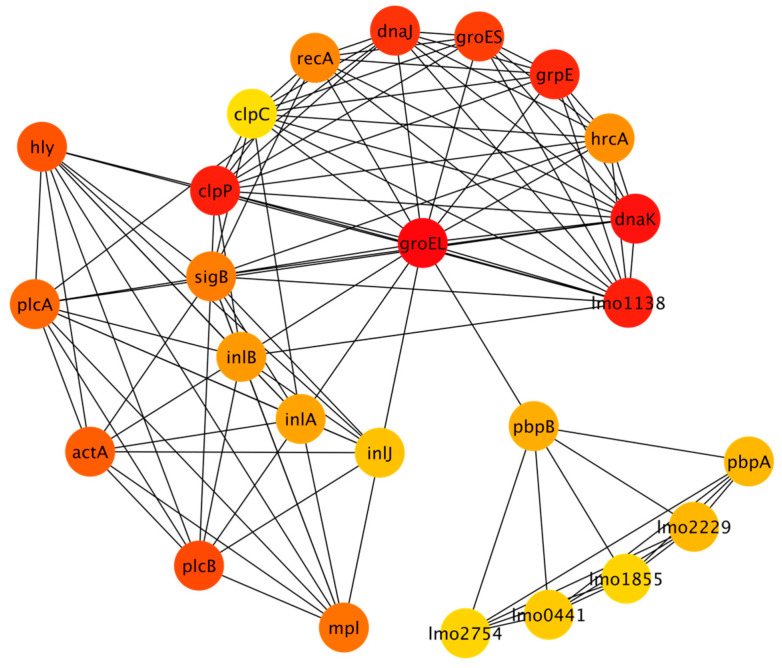
Stress response network depicting top 25 nodes (refer to Table S1 for additional information about each protein).

The full stress response network that was generated resulted in 300 nodes with 1188 edges. The full network is included in the supplemental document Table S1. MCODE analysis identified fourteen clusters ranging in size (from three nodes to forty-four nodes), with a total of one hundred thirty-seven nodes among all the clusters. These nodes were used to generate a reduced STRING network with 137 nodes and 599 edges. The nodes included in the clustered network are also identified in Table S1. CytoHubba was used to rank the top 25 nodes using the MCC algorithm, as discussed in the Methods section. Figure 2 shows the top 25 nodes in a radial layout, with colors indicating each node’s rank; red corresponds to the highest-ranked nodes while yellow corresponds to the lowest.

The elbow method was used to identify the breakpoint in the scores for the top 25 nodes. There were seven nodes that had the highest scores according to the MCC algorithm. The proteins corresponding to these nodes are groEL, dnaK, clpP, lmo1138, grpE, dnaJ, and groES. More details about these proteins are included in Appendix A. Table A1 summarizes the functions of these proteins. Table A2 shows the STRING functional enrichment annotations for the highest-ranked nodes. The Gene Ontological (GO) terms show that the stress response network enriched the genes related to molecular functions and biological processes and the KEGG pathway related to RNA degradation.

### 3.2. Virulence Protein Interaction Network

**Figure 3:**
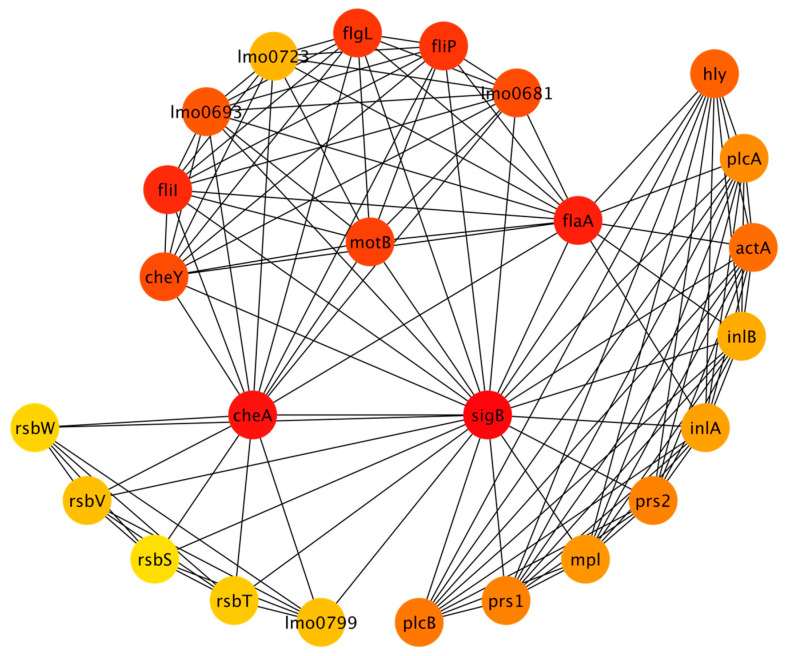
Virulence network depicting top 25 nodes (refer to Table S2 for additional information about each protein).

The full virulence network that was generated resulted in 300 nodes with 1544 edges. The full network is included in the supplemental document Table S2. MCODE analysis identified fourteen clusters ranging in size (from three nodes to thirty-three nodes), with a total of one hundred forty-three nodes among all the clusters. These nodes were used to generate a reduced STRING network with 143 nodes and 840 edges. The nodes included in the clustered network are also identified in Table S2. CytoHubba was used to rank the top 25 nodes using the MCC algorithm. Figure 3 shows the top 25 nodes in a radial layout, with colors indicating each node’s rank; red corresponds to the highest-ranked nodes and yellow corresponds to the lowest.

The elbow method was used to identify the breakpoint in the scores for the top 25 nodes. There were 10 nodes that had the highest scores according to the MCC algorithm. The proteins corresponding to these nodes are sigB, cheA, flaA, fliI, flgL, fliP, motB, cheY, lmo0681, and lmo0693. More details about these proteins are provided in Appendix B. Table A3 summarizes the function of each protein. Table A4 shows the STRING functional enrichment annotations for the highest-ranked nodes. The GO terms show that the virulence network enriched genes related to biological processes, cellular components, and molecular function and the KEGG pathways related to flagellar assembly and bacterial chemotaxis. 

### 3.3. Antibiotic Resistance Protein Interaction Network

**Figure 4:**
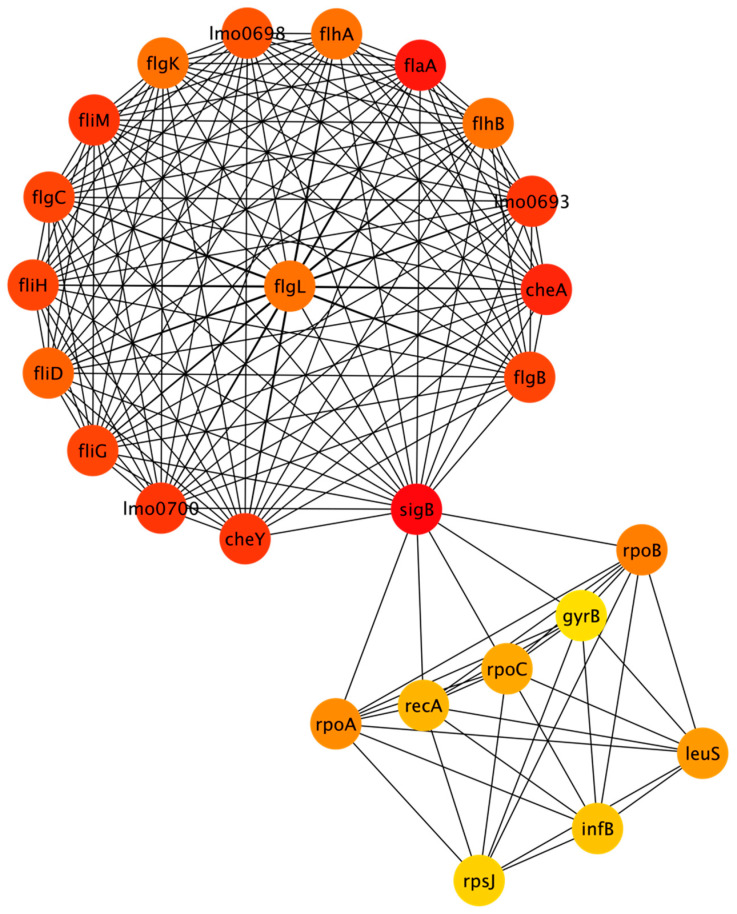
Antibiotic resistance network depicting top 25 nodes (refer to Table S3 for additional information about each protein).

The full antibiotic resistance network that was generated resulted in 300 nodes with 1771 edges. The full network is included in the supplemental document Table S3. MCODE analysis identified sixteen clusters ranging in size (from three nodes to twenty-five nodes), with a total of one hundred fifty-seven nodes among all the clusters. These nodes were used to generate a reduced STRING network with 157 nodes and 1071 edges. The nodes included in the clustered network are also identified in Table S3. CytoHubba was used to rank the top 25 nodes using the MCC algorithm. Figure 4 shows the top 25 nodes in a radial layout, with colors indicating each node’s rank; red corresponds to the highest-ranked nodes and yellow corresponds to the lowest.

The elbow method was used to identify the breakpoint in the scores for the top 25 nodes. There were 17 nodes that had the highest scores according to the MCC algorithm. The proteins corresponding to these nodes are sigB, flaA, cheA, cheY, lmo0693, fliM, lmo0700, flgB, flgC, fliG, fliH, lmo0698, fliD, flhB, flhA, flgK, and flgL. More details about these proteins are included in Appendix C. Table A5 summarizes the function of these proteins. Table A6 shows the STRING functional enrichment annotations for the highest-ranked nodes. The GO terms show that the antibiotic resistance network enriched genes related to biological processes, cellular components, and molecular function and the KEGG pathways related to flagellar assembly and bacterial chemotaxis.

### 3.4. Combined Protein Interaction Network

**Figure 5:**
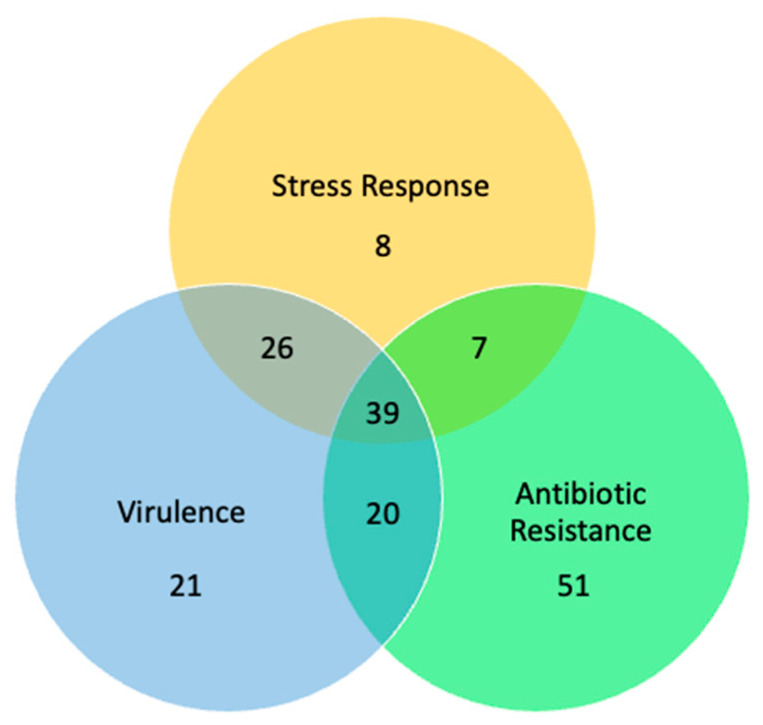
The number of nodes from each individual network, used to create the combined network.

A combined network was generated using the nodes from the top clusters in each of the individual networks. The three individual clustered networks contained one hundred seventy-two unique proteins. The Venn diagram in Figure 5 shows a breakdown of the number of nodes from each individual network that were used to create the combined network.

**Figure 6:**
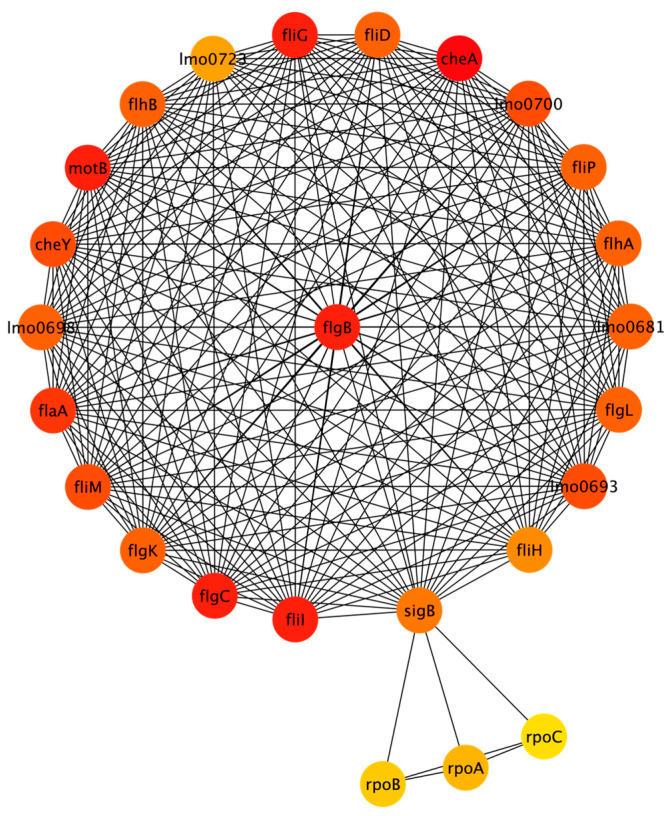
Combined network depicting top 25 nodes (refer to Table S4 for additional information about each protein).

The combined network has 172 nodes and 1429 edges. The full network is included in the supplemental document Table S4. CytoHubba was used to rank the top 25 nodes using the MCC algorithm. Figure 6 shows the top 25 nodes in a radial layout, with colors indicating each node’s rank; red corresponds to the highest-ranked nodes and yellow corresponds to the lowest.

The elbow method was used to identify the breakpoint in the scores for the top 25 nodes. There were 21 nodes that had the highest scores according to the MCC algorithm. The proteins corresponding to these nodes are cheA, flgB, flgC, fliG, fliI, motB, flaA, cheY, fliM, lmo0693, lmo0700, flgK, flgL, flhA, flhB, fliD, fliP, lmo0681, lmo0698, sigB, and fliH. More details about these proteins are included in Appendix D. Table A7 summarizes the function of the protein corresponding to each of these nodes. It also specifies in which individual networks each of the nodes is present. For example, two of the top twenty-one nodes, sigB and flaA, are present in each of the three individual networks. Table A8 shows the STRING functional enrichment annotations for the highest-ranked nodes. The GO terms show that the combined network enriched genes related to biological processes, cellular components, and molecular function and the KEGG pathways related to flagellar assembly and bacterial chemotaxis.

## 4. Discussion

This study outlines a method to generate protein interaction networks using readily available tools and resources. Three individual networks and a combined network were created for stress response, virulence, and antibiotic resistance processes in *Listeria monocytogenes.* Each network was analyzed to identify the most highly interconnected proteins and their functions, and the results were as follows. For the stress response network, the key proteins are groEK, dnaK, lmo1138, clpP, grpE, dnaJ, and groES. The functions of these proteins are summarized in Table A1 and the functional enrichment analysis from STRING is summarized in Table A2. All these proteins have been previously associated with the stress response in *Listeria monocytogenes*: dnak, dnaJ, groEL, groES, and grpE are chaperone proteins involved in the temperature stress response [32,33], and clpP and lmo1138 are involved in the degradation of misfolded proteins in the acid response [34,35].

For the virulence network, the key proteins are sigB, cheA, flaA, fliI, flgL, fliP, motB, cheY, lmo0681, and lmo0693. The functions of these proteins are summarized in Table A3, and the functional enrichment analysis from STRING is summarized in Table A4. Nine of these ten proteins have been previously associated with virulence in *Listeria monocytogenes*: sigB is a sigma factor that contributes to the regulation of virulence gene expression [36]; cheA and cheY are chemotaxis proteins that signal flagellar motors [37]; flaA is the main flagellin protein [38]; fliI, fliP, and flgL are involved in flagellum synthesis [38,39]; motB is involved in motor control [39]; and lmo0681 is a flagellum synthesis regulator [40]. These proteins are primarily involved in motility-related functions, which are known to be virulence factors in bacteria [41].

For the antibiotic resistance network, the key proteins are sigB, flaA, cheA, cheY, lmo0693, fliM, lmo0700, flgB, flgC, fliG, fliH, lmo0698, fliD, flhB, flhA, flgK, and flgL. The functions of these proteins are summarized in Table A5, and the functional enrichment analysis from STRING is summarized in Table A6. Sixteen of these seventeen proteins are involved with chemotaxis and motility-related functions [42] and are not typically associated with antibiotic resistance. However, there are links between these functions and antibiotic resistance. A previous study found that chemotaxis and motility genes are over-expressed in *Listeria monocytogenes* strains in which penicillin-binding and other antibiotic response genes are also over-expressed [42]. The motility-related proteins flaA, flgB, and flgC play a role in biofilm formation and have been shown to be upregulated in response to bactericides [43,44]. Lastly, it has been shown that bacteria in biofilm exhibit increased antibiotic resistance compared to planktonic cells [45]. These observations and the results of this analysis support further studying of the role of these chemotaxis and motility-related proteins in relation to antibiotic resistance. The full antibiotic resistance network, Table S3, also contains multiple known resistance proteins. For example, the results include fosX, which confers fosfomycin resistance [19]; eight pencillin-binding proteins (lmo0441, lmo0550, lmo1438, lmo1855, lmo1916, lmo2229, lmo2754, and lmo2812) and two proteins involved in the regulatory network (fri, lisR), all related to cephalosporin resistance [46]; msrA, which confers macrolide and streptogramin B resistance [47]; and gyrA, lmo2089, and lmo2741, that all confer resistance to fluoroquinolones [47,48]. However, these were not determined to be highly interconnected nodes in the network.

For the combined network, the key proteins are cheA, flgB, flgC, fliG, fliI, motB, flaA, cheY, fliM, lmo0693, lmo0700, flgK, flgL, flhA, flhB, fliD, fliP, lmo0681, lmo0698, sigB, and fliH. The functions of these proteins are summarized in Table A7, and the functional enrichment analysis from STRING is summarized in Table A8. They are generally responsible for motility, chemotaxis, and protein transport and secretion.

Across the three individual networks there are a total of twenty-eight unique proteins (cheA, cheY, clpP, dnaJ, dnaK, flaA, flgB, flgC, flgK, flgL, flhA, flhB, fliD, fliG, fliH, fliI, fliM, fliP, groEL, groES, grpE, lmo0681, lmo0693, lmo0698, lmo0700, lmo1138, motB, and sigB). Two of the highest ranked proteins (sigB and flaA) are present in all three networks. The protein sigB is known to play a role in the regulation of the general stress response and virulence in *Listeria monocytogenes* [7,18,49], and this analysis demonstrates that sigB is also a key protein in the antibiotic resistance network. The protein flaA is a flagellar motility gene involved in biofilm formation [50]. It is highly interconnected in both the virulence and antibiotic resistance networks and is also involved in stress response. Lastly, there are three proteins, cheA, cheY, lmo0693, that are present in the top nodes for the virulence and antibiotic resistance networks.

Prior studies have investigated the key genes and proteins in the stress response [18,49], virulence [7,11,19,51], and antibiotic resistance [26] of various microorganisms. This work analyzes all three processes and their interaction as targets to combat *Listeria monocytogenes.* To the best of the authors’ knowledge, this is the first work to create and analyze a protein interaction network for antibiotic resistance and for the combined processes of stress response, virulence, and antibiotic resistance. In addition, while previous works have described genes and proteins for stress response and virulence, this work expands on those results by identifying a larger network of proteins and the key targets within the network. For example, Hecker et al. identified nine genes (sigB, gadCB, gadD, bsh, opuC, bilE, inlA, inlB, prfA) involved in the stress response in *L. monocytogenes* [18], while this work identifies seven key proteins expressed by different genes. Rantsiou et al., identified seven virulence factors (plcA, iap, hly, prfA, plcB, mpl, and actA) in *L. monocytogenes* [7], while this work identifies ten different key proteins expressed by different genes. 

The results presented here provide the basis for further work to improve food preservation methods to reduce the prevalence of *L. monocytogenes* in food supply; decrease virulence to limit the severity of infections for people exposed to *L. monocytogenes*; and mitigate against the risk of antibiotic resistance in *L. monocytogenes* by identifying new treatments and synergistic compounds to maintain the effectiveness of current treatments for people infected by *L. monocytogenes.* New inhibitors for these target proteins can be evaluated using methods previously described in the literature [8,52]. An improvement in even a single area can have a positive outcome on the control of *L. monocytogenes*. For example, anti-virulence drugs can be developed to target virulence factors and used as alternatives to antibiotic treatments [53,54].

While this analysis has identified several protein targets for further study, there are potential disadvantages to the method. One disadvantage is that results may not include all known key proteins for *Listeria monocytogenes*, or they may not be highly ranked within the network. For example, prfA (lmo0200) is a known bacterial transcription factor that controls the expression of key virulence factors [51], but it was not highly interconnected within the network and therefore not included in the list of key proteins determined via the analysis. Additionally, STRING includes proteins in the network based on direct and indirect interactions in its database. Another disadvantage is that highly ranked proteins included in the network based on indirect interactions may not actually be key proteins for the biological process represented by the network. These disadvantages can be mitigated during the manual curation step by including or excluding specific proteins. Another path for further study of the networks generated in this analysis is to identify key proteins from other studies that were not included. The networks can then be manually curated and analyzed with these proteins included.

## 5. Conclusions

*Listeria monocytogenes* is a deadly and costly foodborne pathogen that is difficult to control and a significant concern for the food industry. Current methods to combat the pathogen can be improved through a better understanding of the processes of stress response, virulence, and antibiotic resistance and their interaction. The key proteins for these processes were determined through the creation and analysis of individual and combined protein interaction networks. Across the three individual networks, twenty-eight key proteins were identified (cheA, cheY, clpP, dnaJ, dnaK, flaA, flgB, flgC, flgK, flgL, flhA, flhB, fliD, fliG, fliH, fliI, fliM, fliP, groEL, groES, grpE, lmo0681, lmo0693, lmo0698, lmo0700, lmo1138, motB, and sigB). While all of these proteins are potential targets for new methods to combat *L. monocytogenes*, five of the twenty-eight proteins (sigB, flaA, cheA, cheY, and lmo0693) represent the most promising targets because they are key proteins in the combined network. These results provide a starting point for further work to identify new strategies to improve food preservation methods and treatments for *L. monocytogenes.*

## Figures and Tables

**Table 1:** STRING settings to generate protein list and create network.

Parameter	Stress	Virulence	Antibiotic Resistance
Data source	STRING: PubMed query	STRING: PubMed query	STRING: PubMed query
Species	*Listeria monocytogenes* EGDe	*Listeria monocytogenes* EGDe	*Listeria monocytogenes* EGDe
Search term	Stress response *Listeria*	Virulence *Listeria*	Antibiotic resistance *Listeria*
Network type	Full STRING network	Full STRING network	Full STRING network
Confidence (score) cutoff	0.40	0.40	0.40
Max. number of proteins	300	300	300
Options	Load enrichment data	Load enrichment data	Load enrichment data

**Table 2:** MCODE settings for clustering analysis.

Parameter	Setting
Find clusters	In whole network
Include loops	No
Degree cutoff	2
Haircut	Yes
Fluff	No
Node density cutoff	N/A
Node score cutoff	0.2
K-score	2
Maximum depth	100

## Data Availability

The data presented in this study are available in the supplementary materials.

## Supplementary Materials

The following supporting information can be downloaded at: https://www.mdpi.com/article/10.3390/microorganisms11040930/s1, Table S1: stress response network; Table S2: virulence network; Table S3: antibiotic resistance network; Table S4: combined network.

## Appendix A

Additional information for highest ranked proteins from the stress response network.

**Table A1:** Summary of top 7 nodes for stress response.

Rank	Node	Function
1	groEL	60 kDa chaperonin; prevents misfolding and promotes the refolding and proper assembly of unfolded polypeptides generated under stress conditions.
2	dnaK	Heat shock 70 kDa protein; acts as a chaperone.
3	lmo1138	ATP-dependent Clp protease proteolytic subunit; cleaves peptides in various proteins in a process that requires ATP hydrolysis; has a chymotrypsin-like activity; plays a major role in the degradation of misfolded proteins; belongs to the peptidase S14 family.
4	clpP	ATP-dependent Clp protease proteolytic subunit; cleaves peptides in various proteins in a process that requires ATP hydrolysis; has a chymotrypsin-like activity; plays a major role in the degradation of misfolded proteins; belongs to the peptidase S14 family.
5	grpE	HSP-70 cofactor; participates actively in the response to hyperosmotic and heat shock by preventing the aggregation of stress-denatured proteins, in association with DnaK and GrpE; it is the nucleotide exchange factor for DnaK and may function as a thermosensor; unfolded proteins bind initially to DnaJ; upon interaction with the DnaJ-bound protein, DnaK hydrolyzes its bound ATP, resulting in the formation of a stable complex; GrpE releases ADP from DnaK; ATP binding to DnaK triggers the release of the substrate protein, thus completing the reaction cycle; several rounds of ATP-dependent interactions between DnaJ, DnaK, and GrpE are required for fully efficient folding.
6	dnaJ	Chaperone protein DnaJ; participates actively in the response to hyperosmotic and heat shock by preventing the aggregation of stress-denatured proteins and by disaggregating proteins, also in an autonomous, DnaK-independent fashion; unfolded proteins bind initially to DnaJ; upon interaction with the DnaJ-bound protein, DnaK hydrolyzes its bound ATP, resulting in the formation of a stable complex; GrpE releases ADP from DnaK; ATP binding to DnaK triggers the release of the substrate protein, thus completing the reaction cycle; several rounds of ATP-dependent interactions between DnaJ, DnaK, and GrpE are required for fully efficient folding; also involved, together with DnaK and GrpE, in the DNA replication of plasmids through the activation of initiation proteins.
7	groES	10 kDa chaperonin; binds to Cpn60 in the presence of Mg-ATP and suppresses the ATPase activity of the latter

**Table A2:** STRING functional enrichment analysis for top 7 nodes for stress response.

Category	Description	Proteins	*p*-Value
GO Molecular Function	Unfolded protein binding	dnaJ, dnaK, grpE, groEL, groES	1.65 × 10^−11^
GO Molecular Function	Heat shock protein binding	dnaJ, dnaK	3.04 × 10^−5^
GO Molecular Function	Chaperone binding	grpE, groES	3.04 × 10^−5^
GO Molecular Function	Protein binding	dnaJ, dnaK, grpE, groEL, groES, clpP	4.91 × 10^−10^
GO Biological Process	Protein folding	dnaJ, dnaK, grpE, groEL, groES	3.36 × 10^−10^
GO Biological Process	Chaperone cofactor-dependent protein refolding	dnaJ, dnaK, groEL, groES	8.61 × 10^−10^
GO Biological Process	Protein refolding	dnaJ, dnaK, groE	1.05 × 10^−6^
KEGG Pathways	RNA degradation	dnaK, grpE, groEL	3.49 × 10^−5^

## Appendix B

Additional information for highest ranked proteins from the virulence network.

**Table A3:** Summary of top 10 nodes for virulence.

Rank	Node	Function
1	sigB	RNA polymerase sigma factor; sigma factors are initiation factors that promote the attachment of RNA polymerase to specific initiation sites and are then released.
2	cheA	Chemotaxis protein CheA; involved in the transmission of sensory signals from the chemoreceptors to the flagellar motors; CheA is autophosphorylated; it can transfer its phosphate group to either CheB or CheY (by similarity).
3	flaA	Flagellin; flagellin is the subunit protein which polymerizes to form the filaments of bacterial flagella.
4	fliI	Involved in type III protein export during flagellum assembly
5	flgL	Lmo0706 protein; with FlgK, acts as a hook filament junction protein to join the flagellar filament to the hook; belongs to the bacterial flagellin family.
5	fliP	FliP, with proteins FliQ and FliR, forms the core of the central channel in the flagella export apparatus.
7	motB	Not available.
8	cheY	Chemotaxis protein CheY; involved in the transmission of sensory signals from the chemoreceptors to the flagellar motors; CheY seems to regulate the clockwise (CW) rotation (by similarity).
8	lmo0681	Lmo0681 protein; positive regulator of class III flagellar genes.
10	lmo0693	Not available.

**Table A4:** STRING functional enrichment analysis for top 10 nodes for virulence.

Category	Description	Proteins	*p*-Value
GO Biological Process	Locomotion	fliP, motB, flaA, cheY, cheA, lmo0693, flgL, fliI	1.48 × 10^−14^
GO Biological Process	Archaeal or bacterial-type flagellum-dependent cell motility	fliP, motB, flaA, cheY, lmo0693, flgL, fliI	1.23 × 10^−12^
GO Biological Process	Bacterial-type flagellum-dependent cell motility	fliP, flaA, lmo0693, flgL, fliI	1.46 × 10^−8^
GO Biological Process	Chemotaxis	motB, cheY, cheA, lmo0693	9.88 × 10^−8^
GO Biological Process	Localization	fliP, lmo0681, motB, flaA, cheY, lmo0693, flgL, fliI	1.76 × 10^−5^
GO Biological Process	Bacterial-type flagellum organization	fliP, lmo0681, fliI	3.89 × 10^−5^
GO Biological Process	Protein transport	fliP, lmo0681, fliI	2.60 × 10^−4^
GO Biological Process	Protein secretion	fliP, fliI	5.90 × 10^−4^
GO Cellular Component	Bacterial-type flagellum	fliP, flaA, lmo0693, flgL	7.58 × 10^−7^
GO Cellular Component	Bacterial-type flagellum basal body	fliP, lmo0693	0.0011
KEGG Pathways	Flagellar assembly	fliP, motB, flaA, lmo0693, flgL, fliI, sigB	3.04 × 10^−12^
KEGG Pathways	Bacterial chemotaxis	motB, cheY, cheA, lmo0693	5.23 × 10^−7^

## Appendix C

Additional information for highest ranked proteins from the antibiotic resistance network.

**Table A5:** Summary of top 17 nodes for antibiotic resistance.

Rank	Node	Function
1	sigB	RNA polymerase sigma factor; sigma factors are initiation factors that promote the attachment of RNA polymerase to specific initiation sites and are then released.
2	flaA	Flagellin; flagellin is the subunit protein which polymerizes to form the filaments of bacterial flagella.
3	cheA	Chemotaxis protein CheA; involved in the transmission of sensory signals from the chemoreceptors to the flagellar motors; CheA is autophosphorylated; it can transfer its phosphate group to either CheB or CheY (by similarity).
4	cheY	Chemotaxis protein CheY; involved in the transmission of sensory signals from the chemoreceptors to the flagellar motors; CheY seems to regulate the clockwise (CW) rotation (by similarity).
4	lmo0693	Not available.
4	fliM	Lmo0699 protein; with FliG and FliN, it makes up the switch complex which is involved in switching the direction of the flagella rotation.
4	lmo0700	One of three proteins involved in switching the direction of the flagellar rotation.
8	flgB	Flagellar basal body rod protein FlgB; structural component of flagellum, the bacterial motility apparatus; part of the rod structure of flagellar basal body.
8	flgC	Flagellar basal-body rod protein FlgC; with FlgF and B, it makes up the proximal portion of the flagellar basal-body rod.
8	fliG	One of three proteins involved in switching the direction of the flagellar rotation.
8	fliH	Lmo0715; binds to and inhibits the function of flagella-specific ATPase FliI
12	lmo0698	One of three proteins involved in switching the direction of the flagellar rotation.
13	fliD	Flagellar hook-associated protein 2; required for morphogenesis and for the elongation of the flagellar filament by facilitating polymerization of the flagellin monomers at the tip of the growing filament; forms a capping structure, which prevents flagellin subunits (transported through the central channel of the flagellum) from leaking out without polymerization at the distal end.
14	flhB	Membrane protein responsible for substrate specificity switching from rod/hook-type export to filament-type export.
14	flhA	Membrane protein involved in the flagellar export apparatus.
14	flgK	Flagellar hook-associated protein 1; with FlgL acts as a hook filament junction protein to join the flagellar filament to the hook.

**Table A6:** STRING functional enrichment analysis for top 17 nodes for antibiotic resistance.

Category	Description	Proteins	*p*-Value
GO Biological Process	Locomotion	flhB, flhA, flaA cheY, cheA, lmo0693, lmo0698, fliM, lmo0700, flgK, flgL, fliD, flgB, flgC, fliG	5.24 × 10^−27^
GO Biological Process	Archaeal or bacterial-type flagellum-dependent cell motility	flhB|, flhA, flaA, cheY, lmo0693, lmo0698, fliM, lmo0700, flgK, flgL, fliD, flgB, flgC, fliG	3.27 × 10^−25^
GO Biological Process	Bacterial-type flagellum-dependent cell motility	flhB, flhA, flaA, lmo0693, lmo0698, fliM, lmo0700, flgK, flgL, fliD, flgB, flgC, fliG	3.58 × 10^−23^
GO Biological Process	Bacterial-type flagellum organization	flhB, flhA, fliM, lmo0700, flgK, flgC, fliG, fliH	3.07 × 10^−13^
GO Biological Process	Chemotaxis	cheY, cheA, lmo0693, lmo0698, fliM, lmo0700, fliG	1.83 × 10^−12^
GO Biological Process	Bacterial-type flagellum assembly	flhB, flhA, fliM, lmo0700, flgK, flgC, fliG	6.63 × 10^−12^
GO Biological Process	Bacterial-type flagellum-dependent swarming motility	flhB, flhA, fliM, lmo0700, flgC, fliG	4.05 × 10^−10^
GO Biological Process	Cellular process	flhB, flhA, flaA, cheY, cheA, lmo0693, lmo0698, fliM, lmo0700, flgK, flgL, fliD, flgB, flgC, fliG, fliH, sigB	1.60 × 10^−4^
GO Cellular Component	Bacterial-type flagellum	flaA, lmo0693, lmo0698, fliM, lmo0700, flgK, flgL, fliD, flgB, flgC, fliG	3.57 × 10^−19^
GO Cellular Component	Bacterial-type flagellum basal body	lmo0693, lmo0698, fliM, lmo0700, flgB, flgC, fliG	4.43 × 10^−12^
GO Cellular Component	Bacterial-type flagellum hook	flgK, flgL, fliD, flgC	2.66 × 10^−7^
GO Cellular Component	Bacterial-type flagellum filament	flaA, fliD	1.90 × 10^−4^
GO Cellular Component	Bacterial-type flagellum basal body, rod	flgB, flgC	1.90 × 10^−4^
GO Cellular Component	Extracellular region	flaA, flgK, flgL, fliD	0.0017
GO Molecular Function	Motor activity	lmo0693, lmo0698, fliM, lmo0700, fliG, fliH	4.55 × 10^−11^
KEGG Pathways	Flagellar assembly	flhB, flhA, flaA, lmo0693, lmo0698, fliM, lmo0700, flgK, flgL, fliD, flgB, flgC, fliG, fliH, sigB	5.24 × 10^−27^
KEGG Pathways	Bacterial chemotaxis	cheY, cheA, lmo0693, lmo0698, fliM, lmo0700, fliG	2.71 × 10^−11^

## Appendix D

Additional information for highest ranked proteins from the combined network.

**Table A7:** Summary of top 21 nodes for the combined network.

Rank	Node	Networks ^1^	Function
1	cheA	V, AR	Chemotaxis protein CheA; involved in the transmission of sensory signals from the chemoreceptors to the flagellar motors; CheA is autophosphorylated; it can transfer its phosphate group to either CheB or CheY (by similarity).
2	flgB	AR	Flagellar basal-body rod protein FlgB; structural component of flagellum, the bacterial motility apparatus; part of the rod structure of flagellar basal-body.
2	flgC	AR	Flagellar basal-body rod protein FlgC; with FlgF and B makes up the proximal portion of the flagellar basal-body rod.
2	fliG	AR	One of three proteins involved in switching the direction of the flagellar rotation.
2	fliI	V	Involved in type III protein export during flagellum assembly.
2	motB	V	Not available.
7	flaA	S, V, AR	Flagellin; flagellin is the subunit protein which polymerizes to form the filaments of bacterial flagella.
8	cheY	V, AR	Chemotaxis protein CheY; involved in the transmission of sensory signals from the chemoreceptors to the flagellar motors; CheY seems to regulate the clockwise (CW) rotation (by similarity).
8	fliM	AR	Lmo0699 protein; with FliG and FliN, it makes up the switch complex which is involved in switching the direction of the flagella rotation.
8	lmo0693	V, AR	Not available.
8	lmo0700	AR	One of three proteins involved in switching the direction of the flagellar rotation.
12	flgK	AR	Flagellar hook-associated protein 1; with FlgL, it acts as a hook filament junction protein to join the flagellar filament to the hook.
12	flgL	V, AR	Lmo0706 protein; with FlgK, it acts as a hook filament junction protein to join the flagellar filament to the hook; belongs to the bacterial flagellin family.
12	flhA	AR	Membrane protein involved in the flagellar export apparatus.
12	flhB	AR	Membrane protein responsible for substrate specificity switching from rod/hook-type export to filament-type export.
12	fliD	AR	Flagellar hook-associated protein 2; required for morphogenesis and for the elongation of the flagellar filament by facilitating polymerization of the flagellin monomers at the tip of the growing filament; forms a capping structure, which prevents flagellin subunits (transported through the central channel of the flagellum) from leaking out without polymerization at the distal end.
12	fliP	V	FliP, with proteins FliQ and FliR, forms the core of the central channel in the flagella export apparatus.
12	lmo0681	V	Lmo0681 protein; positive regulator of class III flagellar genes.
12	lmo0698	AR	One of three proteins involved in switching the direction of the flagellar rotation.
20	sigB	S, V, AR	RNA polymerase sigma factor; sigma factors are initiation factors that promote the attachment of RNA polymerase to specific initiation sites and are then released.
21	fliH	AR	Lmo0715; binds to and inhibits the function of flagella specific ATPase FliI.

^1^ S = stress response network, V = virulence network, AR = antibiotic resistance network.

**Table A8:** STRING functional enrichment analysis for top 21 nodes for the combined network.

Category	Description	Proteins	*p*-Value
GO Biological Process	Locomotion	fliP, flhB, flhA, motB, flaA, cheY, cheA, lmo0693, lmo0698, fliM, lmo0700, flgK, flgL, fliD, flgB, flgC, fliG, fliI	2.04 × 10^−31^
GO Biological Process	Archaeal or bacterial-type flagellum-dependent cell motility	fliP, flhB, flhA, motB, flaA, cheY, lmo0693, lmo0698, fliM, lmo0700, flgK, flgL, fliD, flgB, flgC, fliG, fliI	8.13 × 10^−30^
GO Biological Process	Bacterial-type flagellum-dependent cell motility	fliP, flhB, flhA, flaA, lmo0693, lmo0698, fliM, lmo0700, flgK, flgL, fliD, flgB, flgC, fliG, fliI	1.34 × 10^−25^
GO Biological Process	Bacterial-type flagellum organization	fliP, flhB, flhA, lmo0681, fliM, lmo0700, flgK, flgC, fliG, fliH, fliI	4.02 × 10^−18^
GO Biological Process	Bacterial-type flagellum assembly	fliP, flhB, flhA, fliM, lmo0700, flgK, flgC, fliG, fliI	5.99 × 10^−15^
GO Biological Process	Chemotaxis	motB, cheY, cheA, lmo0693, lmo0698, fliM, lmo0700, fliG	1.24 × 10^−13^
GO Biological Process	Bacterial-type flagellum-dependent swarming motility	fliP, flhB, flhA, fliM, lmo0700, flgC, fliG, fliI	3.31 × 10^−13^
GO Biological Process	Localization	fliP, flhB, flhA, lmo0681, motB, flaA, cheY, lmo0693, lmo0698, fliM, lmo0700, flgK, flgL, fliD, flgB, flgC, fliG, fliI	7.22 × 10^−12^
GO Biological Process	Protein secretion	fliP, flhB, flhA, fliI	1.42 × 10^−6^
GO Biological Process	Protein transport	fliP, flhB, flhA, lmo0681, fliI	7.73 × 10^−6^
GO Biological Process	Cellular process	fliP, flhB, flhA, lmo0681, motB, flaA, cheY, cheA, lmo0693, lmo0698, fliM, lmo0700, flgK, flgL, fliD, flgB, flgC, fliG, fliH, fliI, sigB	1.99 × 10^−5^
GO Cellular Component	Bacterial-type flagellum	fliP, flaA, lmo0693, lmo0698, fliM, lmo0700, flgK, flgL, fliD, flgB, flgC, fliG	9.12 × 10^−20^
GO Cellular Component	Bacterial-type flagellum basal body	fliP, lmo0693, lmo0698, fliM, lmo0700, flgB, flgC, fliG	3.31 × 10^−13^
GO Cellular Component	Bacterial-type flagellum hook	flgK, flgL, fliD, flgC	6.61 × 10^−7^
GO Cellular Component	Bacterial-type flagellum filament	flaA, fliD	3.00 × 10^−4^
GO Cellular Component	Bacterial-type flagellum basal body, rod	flgB, flgC	3.00 × 10^−4^
GO Molecular Function	Motor activity	lmo0693, lmo0698, fliM, lmo0700, fliG, fliH	1.96 × 10^−10^
GO Molecular Function	Nucleoside-triphosphatase activity	lmo0681, lmo0693, lmo0698, fliM, lmo0700, fliG, fliH, fliI	8.98 × 10^−6^
KEGG Pathways	Flagellar assembly	fliP, flhB, flhA, motB, flaA, lmo0693, lmo0698, fliM, lmo0700, flgK, flgL, fliD, flgB, flgC, fliG, fliH, fliI, sigB	2.04 × 10^−31^
KEGG Pathways	Bacterial chemotaxis	motB, cheY, cheA, lmo0693, lmo0698, fliM, lmo0700, fliG	2.49 × 10^−12^
